# Income-Related Inequalities in Informal Care: Evidence From the Longitudinal Healthy Longevity Survey in China

**DOI:** 10.1093/geronb/gbab043

**Published:** 2021-03-11

**Authors:** Yixiao Wang, Wei Yang, Mauricio Avendano

**Affiliations:** 1 Department of Global Health and Social Medicine, King’s College London, UK; 2 Department of Social and Behavioural Sciences, Harvard School of Public Health, Boston, Massachusetts, USA

**Keywords:** China, Functional limitations, Income, Informal care, Older people

## Abstract

**Objectives:**

This report aims to examine income-related inequalities in informal care among older people with functional limitations in China.

**Methods:**

Data are drawn from the 2005, 2008, 2011, and 2014 waves of the Chinese Longitudinal Healthy Longevity Survey. Erreygers concentration index, concentration index, and horizontal inequity index are used to examine inequalities in informal care. A random effects model is then used to investigate the relationship between household income and informal care.

**Results:**

There is no significant association between household income and the probability of receiving informal care. However, we observed a significant positive association between household income and hours of informal care received, indicating that those with higher household income receive more hours of informal care compared to those with lower household income. The degree of this inequality increases as the number of functional limitations increases.

**Discussion:**

Lower household income is associated with lower intensity of informal care received, particularly for older people with more functional limitations. Policies are required to support low-income older people with more functional limitations.

The number of older people with functional limitations has been increasing over the past decades in China. In 2015, approximately 16–41 million older people in China have difficulties in performing basic daily activities, among which more than 98% of people receive care at home (Peng & Wu, 2021). Findings from some high-income countries with well-developed long-term care (LTC) system, such as the Netherlands, suggest that older people with higher income have more financial resources to seek formal paid care, whereas older people with lower income tend to turn to family members for help (García-Gómez et al., 2015; Ilinca et al., 2017). However, a few studies have examined inequalities in care in countries with an underdeveloped LTC system.

China, like many other low- and middle-income countries, is still in the initial stage of LTC development, so care responsibility is mainly reliant on families (Hu & Ma, 2018). However, with rapid socioeconomic development, the meaning of filial piety has been altered from an unconditional duty to take care of older parents to a type of support that is to some extent conditional on older parents’ support to adult children (Cong & Silverstein, 2008). For example, adult children may provide care in expectation of future transfers, including bequests (Pezzin et al., 2015). Moreover, with accelerated urbanization and internal migration over the last decades, adult children with higher income may provide more care because they have more flexibility in time allocation decisions (Liu, 2014; Qian, 2017). Thus, older parents with higher income may receive more informal care, compared to those with lower income.

The severity of functional limitations may also influence informal care receipt differently by income. Low-income older parents with more functional limitations cannot provide sufficient bequests to adult children, yet they often require a higher level of needs for care (Soldo & Hill, 1993). Therefore, older parents with lower income and more functional limitations may be less likely to receive informal care than their higher income counterparts. We examined this hypothesis by assessing whether income-related inequality in informal care is larger when the number of functional limitations increases.

Using data from the 2005 to 2014 waves of the Chinese Longitudinal Healthy Longevity Survey (CLHLS), we first examined how the distribution of informal care varies across income groups. We then examined the relationship between care needs, income, and informal care, to assess whether the inequality in informal care across income increases together with needs measured by functional limitations.

## Method

### Data

We used data from the 2005, 2008, 2011, and 2014 waves of the CLHLS, a nationally representative longitudinal survey of older people in China (Zeng, 2004). We limited the analysis to older people with one or more limitations in activities of daily living (ADL) and excluded individuals (*n* = 442) living in nursing homes from our final sample (*N* = 11,1158; see Supplementary Figure 1 for sample description). Supplementary Table 1 displays descriptive statistics of the sample.

### Dependent Variable: Informal Care

We constructed two dependent variables: (a) LTC receipt, comprising three sets of binary variables: no care received, informal care received as primary source (i.e., primary caregiver is spouse, children, grandchildren, other relatives, friends, or neighbors), and formal community-based care received as primary source (i.e., primary caregiver is social services or housekeepers); (b) intensity of informal care, a continuous variable with logarithmic form, based on the information on number of hours of care received from adult children and grandchildren last week.

### Independent Variables of Interest: Household Per Capita Income

Household income is a continuous variable with logarithmic form, based on the question, “What was the income per capita of your household last year?” Income is inflated to 2014 using consumer price indexes. Household size and demographic composition are taken into consideration to adjust the household income using the Equivalent Scale, following the form: $AE={\left(A+PK\right)}^{F}$, where *A* is the number of adults in the household, *K* is the number of children in the household, *P* is the proportion of a child treated as an adult, and *F* is the scale economy factor. In this study, *P* is 0.3 and *F* is 0.75 (Citro & Michael, 1995; Yang, 2013). Following the convention, the top 0.5% and bottom 0.5% of adjusted household income distribution are trimmed (Jenkins, 2015).

### Control Variables

Following the previous research (Hu & Ma, 2018), we controlled for a set of needs-related variables and variables not related to needs. One of the needs-related variables is the number of ADL limitations, a continuous variable based on the total number of six activities respondents are unable to perform or need help with. Description of how variables are measured is presented in Supplementary Table 2.

### Empirical Strategy

We first used Erreygers concentration index (EI), concentration index (CI), and horizontal inequity index (HI) to estimate inequality in informal care (O’Donnell et al., 2007). We used EI to estimate inequality in the percentage of receiving informal care (Erreygers, 2009). A positive EI indicates that the receipt of informal care is more concentrated among those with higher household income. We used CI to estimate inequality in the intensity of informal care, by comparing the cumulative distribution of hours of informal care received with the cumulative distribution of individuals ranked by household income. A positive CI indicates pro-rich inequality, meaning that hours of informal care are more concentrated among those with higher household income. In order to isolate inequalities driven only by socio-economic factors, the indirect standardization method is used to estimate HI (O’Donnell et al., 2007). A positive HI indicates a pro-rich inequality, after controlling for needs-related factors (see Supplementary Appendix 4 for more details).

A panel data model with random effects is then used to control for both time-invariant and time-variant variables to examine the relationship between household income and informal care in the inferential analysis (see Supplementary Appendix 4for more details).

## Results

Table 1 displays the proportion of respondents receiving informal care and the average number of informal care hours by income quintiles among older people with limitations. The probability of receiving informal care decreases as income increases, while the number of hours of informal care received increases as income increases, particularly for those with three or more ADL limitations.

**Table 1.** Informal Care Receipt by Income Quintiles Among Older People With Limitations in China

	Percentage of receiving informal care (%)^a^			Average number of informal care hours (hours)^b^		
	Total	ADL <3	ADL ≥3	Total	ADL <3	ADL ≥3
Poorest	97.27 (*n* = 2,258)	97.39 (*n* = 1,128)	97.14 (*n* = 1,130)	43.54	30.78	55.51
Second poorest	94.44 (*n* = 2,034)	95.14 (*n* = 1,105)	94.72 (*n* = 943)	46.23	32.03	62.34
Middle	92.74 (*n* = 2,061)	94.32 (*n* = 1,054)	91.15 (*n* = 1,006)	48.66	33.60	63.33
Second richest	92.10 (*n* = 2,056)	92.25 (*n* = 1,044)	91.94 (*n* = 1,011)	54.66	38.44	71.00
Richest	87.51 (*n* = 1,949)	89.83 (*n* = 1,015)	85.11 (*n* = 933)	54.48	36.42	71.99

^a^For the percentage of receiving informal care, the numbers of observations are 11,158 (total.), 5,699 (ADL <3), and 5,459 (ADL ≥3). Values in the cells represent percentages (absolute number). ADL = activities of daily living.

^b^Focusing on those receiving informal care, for average number of informal care hours, the numbers of observations are 10,203 (total), 5,100 (ADL <3), and 5,103 (ADL ≥3).

Table 2 summarizes the results from the EI, CI, and HI. In terms of informal care receipt, the EI and HI are not significant at the .05 significance level, indicating that there is no significant inequality in informal care receipt across income groups. By contrast, there is a significant pro-rich inequality in the intensity of informal care, indicating that higher household income is associated with more hours of informal care received. In addition, the CI and the HI are higher among older people with three or more ADL limitations.

**Table 2.** Socioeconomic Concentration Indices in Informal Care Among Older People With Limitations in China

		Total	ADL <3	ADL ≥3
Receiving informal care	EI	−0.0090	0.0196	−0.0286
	Confidence interval	−0.025 to 0.007	−0.011 to 0.050	−0.040 to −0.017
	HI	−0.0043	0.0044	−0.0130
	Confidence interval	−0.011 to 0.003	−0.009 to 0.018	−0.018 to −0.008
Intensity of informal care	CI	0.0094***	0.0084***	0.0206***
	Confidence interval	0.008 to 0.011	0.008 to 0.009	0.012 to 0.030
	HI	0.0119***	0.0039***	0.0214***
	Confidence interval	0.009 to 0.015	0.003 to 0.004	0.008 to 0.035

*Note:* EI and CI represent inequity indices for actual use, HI represents horizontal inequity, and ADL represents activities of daily living. EI = Erreygers concentration index; CI = concentration index; HI = horizontal inequity index.

***p < .01.

Table 3 presents the results of the random effects model. Models 1 and 2 show that there is no significant relationship between household income and informal care receipt. The interaction between household income and number of ADL limitations is not significant, either. Models 3 and 4 show that higher household income is significantly associated with more hours of informal care received (β = 0.019, *p* < .05). There is also a significant interaction between household income and the number of ADL limitations: each additional limitation in ADL significantly increases the association between household income and hours of care received (β = 0.017, *p* < .01). The results are consistent with robustness checks (Supplementary Table 4).

**Table 3.** Random effects Models Among Older People With Limitations in China

	Receiving informal care^a^		Intensity of informal care^b^	
Variables	Model 1	Model 2	Model 3	Model 4
LN (income)	0.979 (0.064)	1.062 (0.107)	0.019 (0.009)**	0.018 (0.009)**
Limitations in ADLs	2.049 (0.241)***	2.084 (0.254)***	0.237 (0.007)***	0.239 (0.007)***
LN (income) × Limitations in ADLs		1.055 (0.058)		0.017 (0.004)***
Needs-related variables				
Age	1.049 (0.013)***	1.049 (0.013)***	0.011 (0.002)***	0.010 (0.002)***
Male	1.393 (0.305)	1.392 (0.305)	−0.017 (0.029)	−0.016 (0.029)
Self-rated health				
Bad	Ref	Ref	Ref	Ref
Fair	1.045 (0.263)	1.046 (0.264)	0.002 (0.033)	0.004 (0.033)
Good	1.152 (0.307)	1.161 (0.309)	0.029 (0.035)	0.032 (0.035)
Number of chronic diseases	1.111 (0.081)	1.112 (0.081)	0.029 (0.009)***	0.029 (0.009)***
Cognitive function scores	0.986 (0.012)	0.985 (0.012)	−0.009 (0.001)***	−0.009 (0.001)***
Variables not related to needs				
Education attainment				
No education	Ref	Ref	Ref	Ref
Elementary school	1.191 (0.364)	1.194 (0.365)	−0.050 (0.039)	−0.048 (0.039)
Middle school and above	1.438 (1.046)	1.442 (1.049)	0.007 (0.082)	0.012 (0.082)
Marital status				
Married	Ref	Ref	Ref	Ref
Widowed	1.276 (0.357)	1.272 (0.355)	0.235 (0.041)***	0.235 (0.041)***
Other	0.308 (0.154)**	0.304 (0.152)**	0.463 (0.118)***	0.462 (0.118)***
Residence				
City	Ref	Ref	Ref	Ref
Town	1.437 (0.437)	1.441 (0.439)	−0.334 (0.037)***	−0.334 (0.037)***
Rural	1.000 (0.240)	1.000 (0.241)	−0.281 (0.032)***	−0.282 (0.032)***
Coresidence with family members				
No	Ref	Ref	Ref	Ref
Yes	2.539 (0.651)***	2.516 (0.646)***	0.199 (0.050)***	0.191 (0.050)***
Number of surviving children	1.010 (0.052)	1.010 (0.052)	0.007 (0.007)	0.007 (0.006)
Financial assistance from children				
No	Ref	Ref	Ref	Ref
Yes	1.064 (0.284)	1.055 (0.282)	0.023 (0.035)	0.025 (0.035)
Living in the community with care services				
No	Ref	Ref	Ref	Ref
Yes	0.992 (0.251)	0.991 (0.251)	−0.060 (0.032)*	−0.060 (0.032)*
Year				
2005	Ref	Ref	Ref	Ref
2008	1.077 (0.279)	1.081 (0.279)	0.072 (0.033)**	0.075 (0.033)**
2011	1.075 (0.305)	1.079 (0.307)	−0.017 (0.039)	−0.015 (0.039)
2014	1.086 (0.364)	1.094 (0.367)	0.009 (0.046)	0.011 (0.046)
_cons	0.081 (0.107)	0.146 (0.216)	2.168 (0.179)***	2.173 (0.179)***
*N*	11,158	11,158	10,203	10,203

*Note:* Ref = reference; ADL = activities of daily living; _cons, the constant in the regression; LN, natural logarithm of a number.

^a^Models 1 and 2 are results of receiving informal care from random effects multinomial logistic regression models. The reference category is receiving no care. Results of receiving formal care are given in Supplementary Table 3. Values in the cells represent odds ratio (standard error).

^b^Models 3 and 4 are results of random effects linear regression models. Values in the cells represent coefficient (standard error).

***p < .01, **p < .05, *p < .1.

## Discussion

This study examines income-related inequalities in informal care using a nationally representative sample in China. We find that although there is no inequality in the probability of receiving informal care, among those receiving informal care, higher household income is significantly associated with higher intensity of informal care. This pro-rich inequality in the intensity of informal care significantly increases as the number of ADL limitations increases.

Our findings are consistent with prior studies (An, 2019; Liu, 2015; Yan & Xue, 2018) as well as with predictions from the collective model (Alderman et al., 1995), a common theoretical framework used to explain intergenerational support in China. This model proposes that family members have different amounts of “bargaining power” relative to others in the family. The relative bargaining power of family members guides their interactions and is reflected in the family’s allocation of resources (Chiappori, 1992). With rapid modernization, the traditional value of filial piety has been eroded, the bargaining power of older parents in the family has been weakened, and the unconditional willingness of adult children to care for older parents has declined (Cong & Silverstein, 2012). Increasingly, older parents rely on their socioeconomic resources to influence adult children’s caregiving decisions, by providing downward transfers in exchange for receiving informal care (Lloyd-Sherlock et al., 2018). For example, older parents with higher income are more likely to give money or gifts to adult children in exchange for receiving more informal care (An, 2019). Likewise, adult children may provide more care for older parents with higher income given the expectation of future bequests (Liu, 2015). Furthermore, taking care of older parents with more ADL limitations places heavier caregiving burden on adult children (Saraceno, 2010). Therefore, low-income older people with more ADL limitations have relatively lower bargaining power, leading to less likelihood of receiving informal care.

These findings have significant policy implications. LTC policies should prioritize programs that either provide community-based care targeted to poor households or focus on supporting caregivers from low-income groups, for example, through direct funding or economic subsidies. Moreover, a comprehensive LTC insurance system may be required, particularly for low-income individuals with more limitations. Targeting the most vulnerable might contribute to reduce expenses in formal care services, as well as preventing functional decline (Yang et al., 2020).

Some limitations in our study should be considered. First, we did not have information on hours of care received from spouse, siblings, and other family members, which may be important in understanding inequalities in the receipt of care. Second, while we had data on the receipt of either informal or community-based care, we were not able to identify those receiving mixed care, which is common in some areas in China. Third, while our study demonstrates the magnitude of inequality, future research is needed to examine the causal mechanisms underlying associations observed.

## Funding

This work is funded by King’s-China Scholarship Council PhD Scholarship programme.

## Conflict of Interest

None declared.

## Supplementary Material

gbab043_suppl_Supplementary_Materials
