# Supplementary material for: Income-Related Inequalities in Informal Care: Evidence From the Longitudinal Healthy Longevity Survey in China
Source: J Gerontol B Psychol Sci Soc Sci. 2021 Mar 11;76(8):1691–6. doi: 10.1093/geronb/gbab043 (PMC8522470; doi:10.1093/geronb/gbab043)
Supplement: gbab043_suppl_Supplementary_Materials [file gbab043_suppl_supplementary_materials.docx]

**Supplementary Figure 1. ﻿Flow chart of the study sample** ^a^

3,774 older people

Wave 1 (2005)

3,102 older people first interviewed

515 older people survived

Wave 2 (2008)

1,726 older people first interviewed

482 older people survived

Wave 3 (2011)

89 older people survived

24 older people survived

93 older people survived

371 older people survived

982 older people first interviewed

Wave 4 (2014)

Note: ^a^ In the CLHLS, respondents were first asked whether they required assistance in carrying out each activity of daily living (ADL); if they reported that they did, they were then asked to choose their primary caregiver from the following choices: spouse, children, grandchildren, other relatives, friends, neighbours, social services, or housekeepers. If they reported that this was an informal carer (i.e., spouse, children, grandchildren, other relatives, friends or neighbours), they were then asked about the total number of hours they received help in the last week. Information on informal care is independent of information on institutional care in this survey; the research about community-based care (informal care and formal community-based care) and research about institutional care are often independent of each other in LTC research (Hu & Li, 2018; Hu & Ma, 2018; Yang & Tan, 2019); ﻿institutional care relates to different policies and is financed separately in some localities. Thus, we limited analysis to older people with at least one ADL limitation, dropped older people who were missing on income and informal care (N=74), and excluded a number of observations (N = 442) receiving institutional care from our final sample (N = 11,158) to reduce potential bias in our findings.

**Supplementary Table 1. Descriptive characteristics of the sample ^a^**

| **Variables** | **2005** | **2008** | **2011** | **2014** |
| --- | --- | --- | --- | --- |
|  | **Mean (SD)/Percentages** | | |  |
| **LTC** |  |  |  |  |
| No care | 1.25 | 1.00 | 1.18 | 1.29 |
| Receiving formal care | 7.92 | 5.64 | 5.09 | 2.65 |
| Receiving informal care | 90.83 | 93.36 | 93.73 | 96.05 |
| **Weekly hours of informal care ^b^** | 43.04 (46.43) | 53.61 (53.01) | 51.11 (55.99) | 51.71 (56.09) |
| **Household per capita income** | 4298.47 (11536.27) | 7763.82 (10976.91) | 10717.81 (13369.03) | 12756.00(13872.68) |
| **Age** | 94.52 (8.62) | 95.93 (8.03) | 93.81 (9.38) | 92.96 (9.53) |
| **Gender** |  |  |  |  |
| Female | 69.34 | 70.36 | 65.69 | 64.63 |
| Male | 30.66 | 29.64 | 34.31 | 35.37 |
| **Self-rated Health** |  |  |  |  |
| Bad | 45.57 | 50.84 | 41.72 | 43.69 |
| Fair | 27.03 | 23.00 | 29.93 | 31.14 |
| Good | 27.40 | 26.15 | 28.35 | 25.17 |
| **Number of chronic diseases** | 1.67 (1.70) | 1.27 (1.41) | 1.38 (1.56) | 1.34 (1.47) |
| **Number of limitations in ADLs** | 2.80 (1.85) | 2.93 (1.85) | 2.98 (1.90) | 3.01 (1.90) |
| **Cognitive function score** | 14.28 (10.82) | 11.25 (10.51) | 14.56 (10.50) | 14.89 (10.55) |
| **Education attainment** |  |  |  |  |
| No education | 83.21 | 86.38 | 86.27 | 85.75 |
| Elementary school | 13.76 | 10.89 | 11.39 | 11.88 |
| Middle school and above | 3.03 | 2.72 | 2.34 | 2.38 |
| **Marital status** |  |  |  |  |
| Married | 14.31 | 11.34 | 19.00 | 23.04 |
| Widowed | 84.05 | 87.25 | 79.43 | 75.45 |
| Other | 1.64 | 1.41 | 1.57 | 1.51 |
| **Residence** |  |  |  |  |
| City | 34.84 | 28.92 | 23.29 | 19.86 |
| Town | 17.54 | 16.31 | 28.52 | 31.90 |
| Rural | 47.62 | 54.77 | 48.19 | 48.23 |
| **Co-residence with family members** |  |  |  |  |
| No | 7.45 | 6.05 | 9.23 | 10.60 |
| Yes | 92.55 | 93.95 | 90.77 | 89.40 |
| **Number of surviving children** | 3.41 (2.01) | 3.41 (1.95) | 3.71 (1.95) | 3.63 (1.95) |
| **Financial assistance from children** |  |  |  |  |
| No | 12.33 | 11.67 | 24.91 | 24.50 |
| Yes | 87.67 | 88.33 | 75.09 | 75.50 |
| **Availability of community care services** |  |  |  |  |
| No | 88.23 | 87.16 | 66.81 | 61.90 |
| Yes | 11.77 | 12.84 | 33.19 | 38.10 |
| N | 3,774 | 3,617 | 2,297 | 1,470 |

Notes: **^a^** The unit of this study sample is the individual. The study sample is limited to older people with at least one ADL limitation. Mean (SD) is presented for continuous variables, and Percentages is presented for categorical variables. **^b^** For the weekly hours of informal care, the number of the total sample is 10,370. Specifically, the number of the sample in 2005, 2008, 2011, 2014 are 3,264, 3,481, 2,112, 1,346 respectively.

**Supplementary Table 2. Operationalization of control variables**

| **Needs-related variables** | |
| --- | --- |
| Age | Continuous measure of years |
| Gender | Two dummy indicators: Male, Female |
| Self-Rated Health | Three dummy indicators: Bad; Fair; good |
| Number of chronic diseases | The sum of 24 items indicating suffering from the following items: Hypertension; Diabetes; Heart disease; Stroke, cerebrovascular disease; Bronchitis, emphysema, asthma, pneumonia; Pulmonary tuberculosis; Cataracts; Glaucoma; Cancer; Prostate tumour; Gastric or duodenal ulcer; Parkinson’s disease; Bedsore; Arthritis; Dementia; Epilepsy; Cholecystitis, cholelithiasis disease; Blood disease; Rheumatism or rheumatoid disease; Chronic nephritis; Galactoprotein disease; Uterine tumour; Hyperplasia of prostate; or Hepatitis. |
| Number of limitations in ADLs | The sum of 6 items indicating difficulty with the following items: bathing; dressing; going to the toileting; indoor transferring; continence; or eating. |
| Cognitive function | The sum of 30 questions the respondent answered correctly. These questions comprised six dimensions: orientation, registration, naming, attention and calculation, recall, and language. ﻿The validity and reliability of the Chinese MMSE has been verified (Kuang et al., 2017; Lv et al., 2019; Peng & Wu, 2015). ﻿﻿The Cronbach’s reliability coefficients of orientation, registration, calculation, and recall are 0.919, 0.898, 0.938, 0.892, and 0.907 respectively. |
| **Variables not related to needs** | |
| Education | Three dummy indicators: No education; Elementary school; Middle school and above |
| Marital status | Three dummy indicators: Married; Widowed; Other (separated, divorced, and never married) |
| Residence | Three dummy indicators: City; Town; Rural areas. |
| Living arrangement | Two dummy indicators: Living with family members; Not living with family members |
| Number of surviving children | ﻿An indicator for the number of surviving children an individual had |
| The availability of financial assistance from children | Two dummy indicators: Receiving financial assistance from children; Not receiving financial assistance from children |
| The availability of community care services | Two dummy indicators: Living in the community with community care services; Not living in the community with community care services |

**Supplementary Appendix 4**

Erreygers’s Concentration Index (EI), Concentration Index (CI) and Horizontal Inequity Index (HI), commonly applied indicators to measure the direction and degree of inequality in care use (García-gómez et al., 2015; O’Donnell et al., 2007; Vincenzo et al., 2017), are used to estimate the direction and degree of income-related inequality in informal care in descriptive analysis. The method involves four steps: (1) estimating a model on the determinants of informal care, using need and non-need variables; (2) calculating the unstandardized EI and CI; (3) using the sample mean for variables not related to needs, calculating need-adjusted EI and CI; and (4) estimating HI, showing inequalities in informal care only driven by socioeconomic factors. Because the first dependent variable, whether receiving informal care or not, is a categorical variable, we use EI, ﻿the corrected version of the concentration index for bounded variables suggested by Erreygers, to have a better estimation in inequality in the percentage of receiving informal care (O’Donnell et al., 2007). Because the second dependent variable, intensity of informal care, is a continuous variable, we use CI to compare the cumulative distribution of hours in informal care received with the cumulative distribution of older people ranked by income, to estimate inequality in intensity of informal care.

Specifically, we estimate the CI of informal care as follows:

$CI=\frac{2}{\mu}cov(y_{it},R_{it})$ (1)

Where $\mu$ represents the mean of the dependent variable in the total sample; $y_{it}$ represents the dependent variable; and $R_{it}$ represents the individual’s rank within the income distribution in each year. A positive CI means that there is pro-rich inequality, i.e., informal care is more concentrated among the rich, while a negative CI implies pro-poor inequality, i.e., informal care is more concentrated among the poor. In terms of values of concentration index, an index of 0.9 indicates a pro-rich inequality; compared with an index of 0.1, an index of 0.9 indicates a more pronounced pro-rich inequality for the dependent variable assessed.

﻿However, if the dependent variable of interest is categorical, then the bounds of the CI depend on the mean of the dependent variable (Wagstaff, 2005). The bounds turn out to be wider for populations with a low mean (i.e. close to 0) than for populations with a high mean (i.e. close to 1). Therefore, we use Erreygers’s Concentration Index as follows:

$EI=\frac{4\mu}{(b_{n}-a_{n})}CI$ (2)

﻿Where $b_{n}$ and $a_{n}$ represent the maximum and minimum of the dependent variable of interest, $\mu$ is the mean of the health variable in the population, and $CI$ represents the $CI$ specified in (1). A positive EI indicates the receipt of informal care is more concentrated among the rich.

The inequalities CI and EI show are driven by both need factors and non-need/socioeconomic factors. In order to identify only socioeconomic-related differences in care use, the direct and indirect method of standardization could be used (Kakwani, Wagstaff, & Van Doorslaer, 1997). Due to the fact that the direct standardization requires the use of grouped data, we use indirect standardization to calculate inequalities in informal care only driven by socioeconomic factors. The whole process is as following. First, a model of the determinants of informal care intensity was estimated,

$y_{i}=\alpha+\sum_{k} \beta_{k}N_{ki}+\sum_{j} \gamma_{j}Z_{ji}+\varepsilon_{i}$ (3)

where $y_{i}$ represents the actual use of informal care, $N_{k}$ represents a set of needs-related factors, and $Z_{j}$ represents a set of factors not related to needs.

The needs-adjusted utilisation is then defined as follows:

$\hat{y}_{i}=\hat{\alpha}+\sum_{k} \hat{\beta}_{k}N_{ki}+\sum_{j} \hat{\gamma}_{j}\bar{Z}_{ji}+\varepsilon_{i}$ (4)

where $\hat{y}_{i}$ represents the predicted value of use of informal care. As the equation shows, the actual values of needs-related variables are used for standardisation, while the mean value of variables not related to needs are used as controls.

The indirectly standardised needs-adjusted utilisation is calculated using the difference between actual use of informal care ($y_{i}$) and the predicted value of use of informal care ($\hat{y}_{i}$), adding the sample mean value of use of informal care $\bar{y}$, to find the distribution of use of informal care only associated with factors not related to needs. Thus, a positive HI indicates pro-rich inequality, while a negative HI indicates pro-poor inequality, after controlling for needs-related factors.

Panel data model with random effects as follows is then used to control for both time-invariant and time-variant variables to examine more clearly the relationship between income and informal care in inferential analysis.

$\mathrm{IFC}_{\mathrm{it}}=\alpha_{0}+\alpha_{1}ln({Income)}_{\mathrm{it}}+\alpha_{2}A_{i}+\alpha_{3}B_{\mathrm{it}}+\varepsilon_{\mathrm{it}}$ (5)

where $\mathrm{IFC}_{it}$ represents the percentage or hours of informal care the individual received last week. $A_{i}$represents time-invariant variables (gender and education). $B_{\mathrm{it}}$ represents time-variant variables (other covariates).

To examine the heterogenous relationship between income, informal care and limitations, an interaction of income and number of ADL limitations is added to the model:

$\mathrm{IFC}_{\mathrm{it}}=\alpha_{0}+\alpha_{1}ln({Income)}_{\mathrm{it}}+\alpha_{2}{ln({Income)}_{\mathrm{it}}*Number of ADL limitations}_{\mathrm{it}}+\alpha_{3}{Number of ADL limitations}_{\mathrm{it}}+\alpha_{4}A_{i}+\alpha_{5}B_{\mathrm{it}}+\varepsilon_{\mathrm{it}}$ (6)

A positive value for $\alpha_{2}$ would imply that the more limitation the ADL, the

greater the effect of income on informal care is.

We run random effects multinomial logistic regression models when dealing with the first dependent variable, LTC receipt. We try to include all results of this random effects multinomial logistic regression model in Table 3 in the text, but the table would be too large to present. We could not provide a new table to present the model because the maximum data element in a research report is 3. Finally, we present the results of whether receiving informal care in the paper to show the main results, present the results of whether receiving formal care in Supplementary Table 3.

**Supplementary Table 3 Random effects regression models** ^a^

|  | **Receiving formal care** | |
| --- | --- | --- |
| **Variables** | **Model 1** | **Model 2** |
| **LN (income)** | 1.590 (0.126) *** | 1.720 (0.189) *** |
| **Limitations in ADLs** | 2.620 (0.317) *** | 2.655 (0.333) *** |
| **LN (income) * limitations in ADLs** |  | 1.062 (0.062) |
| **Needs-related variables** |  |  |
| Age | 1.072 (0.015) *** | 1.072 (0.015) *** |
| Male | 1.225 (0.299) | 1.225 (0.298) |
| Self-rated health |  |  |
| Bad | Ref | Ref |
| Fair | 0.900 (0.252) | 0.902 (0.253) |
| Good | 1.073 (0.316) | 1.081 (0.318) |
| Number of chronic diseases | 1.145 (0.088) * | 1.146 (0.088) * |
| Cognitive function scores | 1.003 (0.014) | 1.003 (0.014) |
| **Variables not related to needs** |  |  |
| Education attainment |  |  |
| No education | Ref | Ref |
| Elementary school | 1.729 (0.563) * | 1.735 (0.566) * |
| Middle school and above | 3.998 (2.973) * | 4.012 (2.984) * |
| Marital status |  |  |
| Married | Ref | Ref |
| Widowed | 1.290 (0.414) | 1.285 (0.412) |
| Other | 0.413 (0.277) | 0.409 (0.275) |
| Residence |  |  |
| City | Ref | Ref |
| Town | 0.632 (0.206) | 0.634 (0.207) |
| Rural | 0.135 (0.037) *** | 0.135 (0.037) *** |
| Co-residence with family members |  |  |
| No | Ref | Ref |
| Yes | 0.882 (0.259) | 0.872 (0.256) *** |
| Number of surviving children | 1.029 (0.058) | 1.029 (0.058) |
| Financial assistance from children |  |  |
| No | Ref | Ref |
| Yes | 1.419 (0.419) | 1. 408 (0.416) |
| Living in the community with care services |  |  |
| No | Ref | Ref |
| Yes | 1.284 (0.354) | 1.283 (0.354) ** |
| Year |  |  |
| 2005 | Ref | Ref |
| 2008 | 0.512 (0.145) ** | 0.513 (0.146) ** |
| 2011 | 0.370 (0.118) *** | 0.371 (0.118) *** |
| 2014 | 0.191 (0.075) *** | 0.192 (0.076) *** |
| _cons | 0.000 (0.000) *** | 0.000 (0.000) *** |
| N | 11,158 | 11,158 |

Notes: ^a^ Model 1 and 2 are results of receiving formal care from random effects multinomial logistic regression models. The reference category is receiving no care. Cell represent odds ratio (standard error). *** p<0.01, ** p<0.05, * p<0.1.

In Supplementary Table 4, we replace the number of ADL limitations with a dummy that indicates whether the individual have three or more limitations in ADL, which is consistent with other literature (Anderson et al., 1998; Dunlop, 2002; Dunlop et al., 2001; Ferrucci et al., 1996; Mor et al., 1994; Shen et al., 2015). It is widely agreed that if the individual reported need for help with or inability to perform three or more ADLs, he/she is severely impaired in ADLs. In addition, the report of three or more ADL limitations is of particular interest in LTC research because this level of limitation is often used in many countries to determine whether an older person is eligible for receiving formal LTC (Dunlop et al., 2002), thus we use this binary variable. As Supplementary Table 4 shows, we observe a significantly positive interaction between income and this binary variable. Results from the model suggested that for those with 3 or more limitations in ADL, an increase in household income is significantly associated with a higher increase in number of hours of informal care ($\beta$=0.068, *p*<0.01).

**Supplementary Table 4 Random effects linear models among older people with limitations who receive informal care in China ^a^**

| **Variables** | **Model 1** | **Model 2** |
| --- | --- | --- |
| **Ln (income)** | 0.020 (0.009) ** | -0.014 (0.012) |
| **>=3 limitations in ADLs** | 0.774 (0.027) *** | 0.783 (0.027) *** |
| **Ln (income) * >=3 limitations in ADLs** |  | 0.068 (0.016) *** |
| **Needs-related variables** |  |  |
| Age | 0.010 (0.002) *** | 0.010 (0.002) *** |
| Male | -0.010 (0.029) | -0.008 (0.029) |
| Self-rated health |  |  |
| Bad | Ref | Ref |
| Fair | -0.031 (0.033) | -0.029 (0.033) |
| Good | -0.010 (0.035) | -0.005 (0.035) |
| Number of chronic diseases | 0.036 (0.009) *** | 0.037 (0.009) *** |
| Cognitive function scores | -0.013 (0.001) *** | -0.013 (0.001) *** |
| **Variables not related to needs** |  |  |
| Education attainment |  |  |
| No education | Ref | Ref |
| Elementary school | -0.027 (0.039) | -0.025 (0.039) |
| Middle school and above | 0.017 (0.083) | 0.020 (0.029) |
| Marital status |  |  |
| Married | Ref | Ref |
| Widowed | 0.246 (0.042) *** | 0.245 (0.042) *** |
| Other | 0.499 (0.119) *** | 0.498 (0.119) *** |
| Residence |  |  |
| City | Ref | Ref |
| Town | -0.344 (0.038) *** | -0.343 (0.037) *** |
| Rural | -0.293 (0.033) *** | -0.295 (0.032) *** |
| Co-residence with family members |  |  |
| No | Ref | Ref |
| Yes | 0.219 (0.051) *** | 0.210 (0.051) *** |
| Number of surviving children | 0.007 (0.007) | 0.007 (0.007) |
| Financial assistance from children |  |  |
| No | Ref | Ref |
| Yes | 0.016 (0.035) | 0.019 (0.035) |
| Living in the community with care services |  |  |
| No | Ref | Ref |
| Yes | -0.048 (0.032) | -0.050 (0.032) * |
| Year |  |  |
| 2005 | Ref | Ref |
| 2008 | 0.064 (0.033) * | 0.066 (0.033) ** |
| 2011 | 0.016 (0.039) | 0.004 (0.039) |
| 2014 | 0.012 (0.046) | 0.013 (0.046) |
| _cons | 1.858 (0.181) *** | 1.860 (0.181) *** |
| N | 10,203 | 10,203 |

Notes: ^a^ Cells represent coefficient (standard error). *** p<0.01, ** p<0.05, * p<0.1.
